# Zona incerta: from Parkinson's disease to addiction

**DOI:** 10.3389/fncir.2025.1537449

**Published:** 2025-02-06

**Authors:** Mylène Wilt, Robin Magnard, Sebastien Carnicella, Yvan M. Vachez

**Affiliations:** ^1^Inserm, U1216, Univ. Grenoble Alpes, Grenoble Institut Neurosciences, Grenoble, France; ^2^Department of Psychological and Brain Sciences, Krieger School of Arts and Sciences, Johns Hopkins University, Baltimore, MD, United States

**Keywords:** zona incerta (ZI), addiction, Parkinson's disease, dopamine, deep brain stimulation

## Introduction

**Figure 1:**
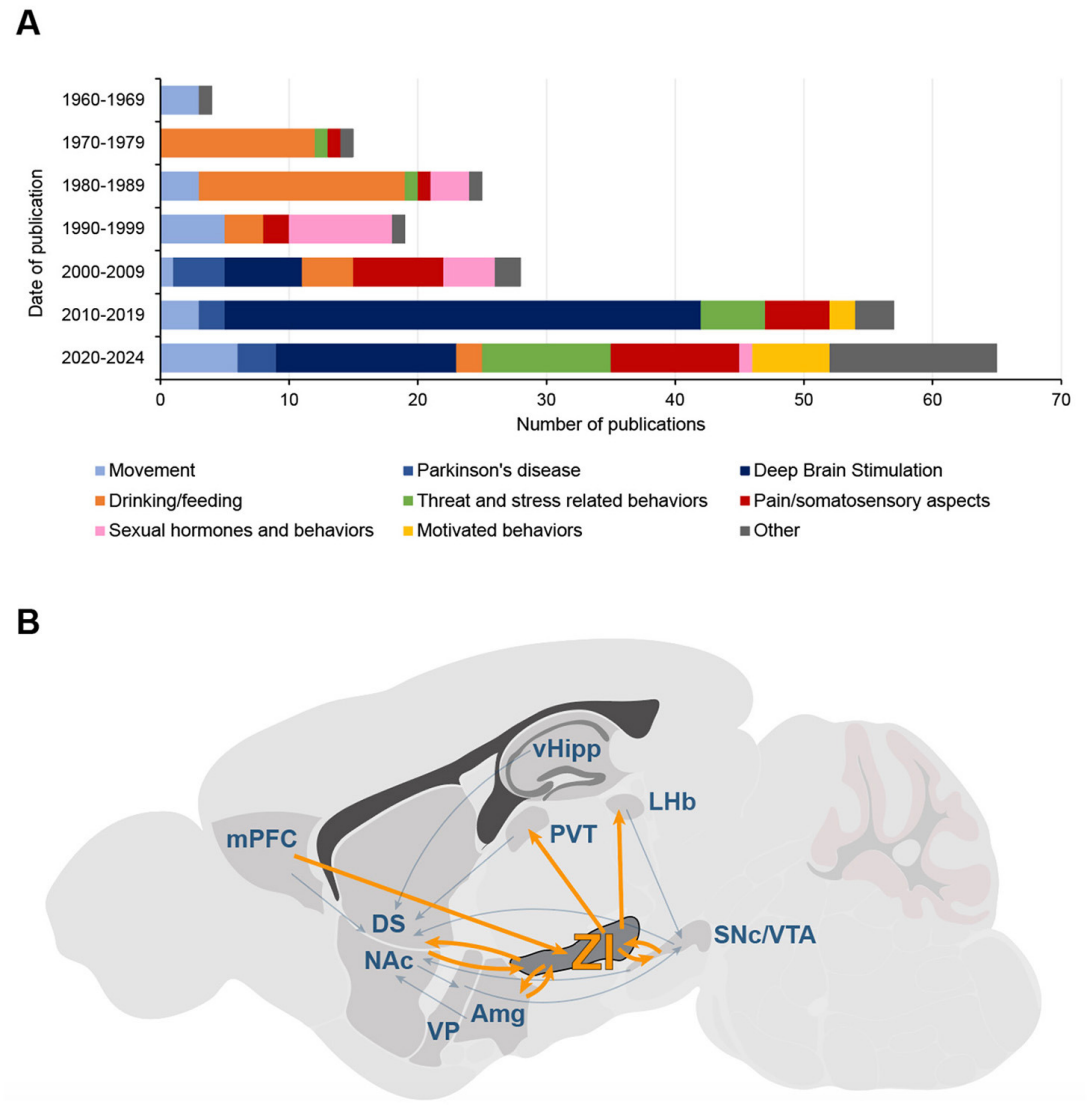
The Zona Incerta interest's evolution, from motor function to addiction? **(A)** Evolution of functions and topics studied in publications on the zona incerta (ZI) from 1965 to 2024. Data were obtained from a bibliometric analysis of scientific literature on PubMed, including only articles with “zona incerta” in the title; studies primarily focused on tracing, anatomy, cytoarchitecture, or neurodevelopment were excluded, even if they referenced functions (*n* = 213 articles). Bars represent the number of publications per decade, divided into main functional and thematic categories: motor functions (light blue), Parkinson's disease (PD; medium blue), deep-brain stimulation (DBS; dark blue), drinking and feeding behaviors (orange), threat and stress-related behaviors (green), pain and somatosensory functions (red), sexual hormones and behaviors (pink), motivated and appetitive behaviors (yellow), other functions (gray). While early research emphasized non-motor functions of the ZI, particularly ingestive behaviors, the focus started to shift to motor-related roles in the 1990s with studies on PD, and even more in the 2010s with a tremendous increase of interest on DBS; in the last years, there has been a resurgence of interest in non-motor functions, including motivated behaviors. **(B)** Position of the zona incerta in the canonical circuits for addiction. The main structures and circuits mediating positive and negative reinforcement in addiction are represented; simplified and adapted from Lüscher and Janak (2021). The zona incerta shares connections with most of these structures, positioning it as a critical node for different aspects of addiction. Amg, amygdala; DS, dorsal striatum; NAc, nucleus accumbens; LHb, lateral habenula; mPFC, medial prefrontal cortex; PVT, paraventricular nucleus of thalamus; SNc, substantia nigra pars compacta; vHipp, ventral hippocampus; VP, ventral pallidum; VTA, ventral tegmental area; ZI, zona incerta.

The zona incerta (ZI), a small and historically overlooked structure located beneath the thalamus, has been increasingly recognized for its role in diverse behavioral processes. Early studies in the 1970s and 1980s explored its role in ingestive behaviors, including drinking and feeding (for review, see Mitrofanis, 2005) (Figure 1A), suggesting its involvement in motivation and survival mechanisms related to food and water intake. The ZI is now known to be a heterogeneous nucleus, divided into four major sectors: rostral (ZIr), dorsal (ZId), ventral (ZIv), and caudal (ZIc) (Mitrofanis, 2005), each with distinct neurochemical profiles. GABAergic cells, which constitute the majority of the ZI's neurons, and parvalbumin positive neurons are mostly concentrated in the ZIv, while the ZId is rich in glutamatergic cells, and the ZIr contains dopaminergic neurons (Mitrofanis, 2005).

While motor functions of the ZI were identified as early as the 60s (Hyde and Toczek, 1962), the focus really shifted on those motor-related roles in the 1990s, as the ZI became studied in the context of Parkinson's disease (PD) (Shi et al., 2024). The introduction of deep brain stimulation (DBS) as a treatment for PD patients, with the subthalamic nucleus (STN) as a primary target (Benabid et al., 2009), revealed that stimulating the nearby ZI also produced significant improvements in motor symptoms (Voges et al., 2002; Ossowska, 2020). This led to a renewed interest for the ZI, placing it in the spotlight for movement disorder therapies. Consequently, its non-motor functions, particularly the potential reward processes driving the ingestive behaviors previously characterized, received less attention. However, reports of non-motor effects following ZI stimulation in PD patients—especially on mood (Stefurak et al., 2003; Tommasi et al., 2008), or emotion (Burrows et al., 2012)—suggested a broader role for the ZI. Notably, motivational alterations such as apathy (Czernecki, 2005; Ricciardi et al., 2014) drew attention to its potential role in motivational processes.

Today, there is a renewed interest in the link between ZI and behaviors related to reward and motivation. The contemporary approaches to map, monitor and manipulate neural circuits are improving our understanding of the contribution of the different subregions and neuronal populations constituting the ZI to its various functions. Notably, the ZI shares remarkable similarities with the substantia nigra pars compacta (SNc) and the ventral tegmental area (VTA), particularly in terms of neuronal populations and their specific involvements in behavior (Mitrofanis, 2005; Morales and Margolis, 2017). While the VTA, and more recently the SNc, are intensively investigated in the context of motivation-related pathologies, especially addiction, this aspect of the ZI remains largely unexamined. Addiction is a chronic psychiatric disorder defined by a loss of control over specific behaviors such as drug intake, despite negative consequences. It involves recurrent periods of drug seeking, withdrawal and relapse, leading to a spiraling addiction cycle (Koob and Le Moal, 2008; Koob and Volkow, 2010). Recent findings on the ZI raise new questions about its potential involvement in addiction related behaviors. This short essay examines current evidence suggesting the ZI's potential role in psychiatric disorders akin to drug addiction and highlights future research directions.

## ZI activity is modulated by drugs of abuse

While the role of ZI in addiction remains underexplored, early pharmacological studies support its responsiveness to drugs of abuse. *In situ* alcohol injection into the ZI elicits strong neuronal responses (Wayner et al., 1975), and injections of tetrahydropapaveroline, previously used to initiate alcohol consumption in rats when injected into the mesocorticolimbic pathway, also induces alcohol preference and consumption when injected into the ZI (Myers and Privette, 1989). Cocaine intake impacts the ZI as well, increasing Fos expression (Zahm et al., 2010) and reducing neurotensin binding, paralleling effects in mesocorticolimbic pathways (Pilotte et al., 1991). Furthermore, morphine induces tyrosine hydroxylase expression and enhances dopamine (DA) metabolism in the ZI, implicating it in dopaminergic modulation (Molnar et al., 1994). These findings indicate that drugs of abuse directly impact ZI activity, supporting its relevance in addiction research.

## ZI dopaminergic neurons mediate motivation

The ZI shares critical features with the VTA and the SNc. A first notable parallel between the three structures is the presence of dopaminergic neurons. Dopaminergic transmission from the VTA and the SNc is instrumental for motivational processes and at the core of addiction disorders. All drugs of abuse trigger DA release in the striatum, particularly in the nucleus accumbens (NAc) (Di Chiara and Imperato, 1988), signaling positive and negative values and valence of biologically relevant stimuli (Bromberg-Martin et al., 2010; Schultz, 1998), but also encoding reward prediction errors (RPE) to guide behavior and learning (Hollerman and Schultz, 1998; Schultz, 1998), making mesolimbic and nigrostriatal DA fundamental for motivated behaviors (Berke, 2018). Alterations in these pathways contribute to drug addiction-like behaviors, from the drug withdrawal symptoms to excessive drug seeking and taking (Keiflin and Janak, 2015; Goutaudier et al., 2023; Giuliano et al., 2019; Koob and Le Moal, 1997). Very few studies have focused on the dopaminergic neurons of the ZI (ZI_DA_), but recent findings suggest a pivotal role for motivation (Ye et al., 2023), as VTA and SNc dopaminergic neurons.

Specifically, ZI_DA_ are overactive during energy deficit, promoting motivation for food-seeking and maintaining food-seeking behavior, partly through projections to the paraventrical nucleus of the thalamus (PVT) (Ye et al., 2023). Moreover, ZI_DA_ also seem to be involved in other appetitive behaviors such as self grooming (Jiang et al., 2024). Increased grooming has been observed during abstinence, reflecting sociability impairments in drug abuse models (Lalanne et al., 2017; Homberg et al., 2002) or as a stereotypical behavior induced by psychostimulants and exacerbated during drug-sensitization (Kalueff et al., 2016). Excessive grooming is also a marker of compulsivity in rodent models of obsessive-compulsive disorders (Mondragón-González et al., 2024). This is consistent with recent connectivity data from both human and non-human primates that have highlighted the ZI as a potential target for treating obsessive-compulsive disorders (ICDs) (Haber et al., 2023).

## ZI GABA neurons activation favor appetitive behavior and novelty seeking

In addition to their well-studied dopaminergic neurons, the VTA and SNc also contain substantial GABAergic populations, another feature in common with the ZI. Similar to VTA GABAergic neurons (Zhou et al., 2022; Lowes et al., 2021), GABAergic neurons of the ZI (ZI_GABA_) regulate reward motivation and appetitive behaviors, and project to the NAc (Wang et al., 2020). Their optogenetic stimulation has been shown to provoke binge-like eating in mice, while their ablation decreases food intake in the long-term, with a reduction of body weight as a result (Zhang and Van Den Pol, 2017). Other types of motivated behaviors have been shown to involve ZI_GABA_, for example hunting behavior (Zhao et al., 2019), but also novelty seeking (Monosov et al., 2022; Ogasawara et al., 2022), a trait of particular interest in the field of drug addiction, as it is an endophenotype of vulnerability, predicting the transition from controlled to compulsive cocaine use (Belin and Deroche-Gamonet, 2012). Optogenetic activation of ZI_GABA_ has been shown to favor exploration of new objects and conspecifics (Ahmadlou et al., 2021). As NAc-projecting VTA GABAergic neurons undergo significant activity changes and synaptic plasticity following drug exposure (Friend et al., 2021; Williams et al., 2018), and contribute to drug motivation (Ting-A-Kee et al., 2013; Elum et al., 2024; Merkel et al., 2024), one can hypothesize that neuroadaptations within the ZI_GABA_-NAc circuit may similarly underlie various aspects of drug-seeking behavior, notably through disinhibition and exploratory behavior toward novel elements in the environment.

## The ZI regulates negative affect

Collectively, the recent studies exposed above highlight the role of the ZI in motivated behaviors, and point out a potential contribution for the positive reinforcement that drives the early phases of the addiction cycle and eventually leads to compulsive drug seeking and taking (Koob and Le Moal, 1997). The later phases of this cycle are marked by periods of abstinence during which individuals experience dysphoria, apathy, anxiety, depression and pain. These withdrawal symptoms likely contribute to craving, compulsive drug use and relapse, through a negative reinforcement process whereby drug is sought and consumed to alleviate the negative affect experienced (Koob and Le Moal, 1997; Robbins et al., 2024). Beyond its role in motivation, the ZI has been shown to be involved in behaviors reminiscent of this negative state, as anxiety (Li et al., 2021), and pain (Li et al., 2023; Lu et al., 2021). Moreover, the ZI is also implicated in aversive learning (Zhou et al., 2021), partly through connections between ZI_DA_ and the amygdala (Zhang et al., 2022), and through thalamic and periaqueductal gray modulation by ZI_GABA_ (Chou et al., 2018; Venkataraman et al., 2021). Thus, the multifaceted functions of the ZI posit it as a potential significant contributor to both positive and negative reinforcement in addiction.

## Integrating the ZI in canonical circuits of addiction

Taken together, all these findings indicate that the ZI may contribute to various aspects of addiction, from withdrawal-related negative symptoms to compulsive drug-seeking, a role that may become increasingly evident when positioning the ZI within the canonical addiction circuit (Lüscher and Janak, 2021) (Figure 1B). In addition to its structural similarities with the VTA and SNc, the ZI shares connections to these two major structures of addiction circuits. It is bidirectionally connected to the VTA and, to a lesser extent, the SNc and projects to the NAc, making it ideally situated to modulate dopaminergic transmission between the midbrain and the striatum (Arena et al., 2024). As mentioned above, the ZI also projects to the PVT (Ye et al., 2023), a key player in motivational circuits that receives inputs from the prefrontal cortex and lateral hypothalamus (Iglesias and Flagel, 2021). The PVT projects to the NAc and has been implicated in drug addiction, driving drug-seeking behavior and withdrawal symptoms (Zhou and Zhu, 2019). While the VTA and SNc do not provide dopaminergic input to the PVT (Li et al., 2014), the ZI could play this modulatory role, linking homeostatic and reward-driven processes, and modulating the activity of the NAc both directly and indirectly. This aligns with the observed decrease in striatal DA metabolism after ZI electrolytic lesion in rats (Walker et al., 2010). Finally, the ZI is connected to several other nuclei involved in positive and negative reinforcement circuits for addiction, including the prefrontal cortex, amygdala, and lateral habenula (Arena et al., 2024) (Figure 1B). Thus, the ZI is strategically positioned to modulate numerous canonical circuits implicated in addiction processes.

## Discussion

Overall, while clear evidence is still emerging, the ZI's connectivity with addiction-related circuits, including dopaminergic pathways, and its role in motivation, emotion, and interoception, provide strong theoretical support for its role in addiction. The presence of ZI_DA_ potentially recapitulating to a certain extent some feature of VTA/SNc dopaminergic neurons is obviously a strong argument underpinning this opinion (Jiang et al., 2024). However, the precise involvement of ZI_DA_ is discussed, as others found no alteration of motivated behaviors when manipulating this cell population, and highlighted a role limited to prehensile movement (Garau et al., 2023). This is in stark contrast with the place preference induced by ZI_DA_ optogenetic activation (Jiang et al., 2024), for instance. Furthermore, while the involvement of ZI_GABA_ in motivation seems to be more widely accepted, the clear distinction between ZI GABA and DA transmission must however be put into perspective, as it seems that ZI_DA_ are not purely dopaminergic but also co-express GABA neurotransmission markers (Negishi et al., 2020). Beyond these considerations, several key questions remain unanswered: Does drug intake induce DA release from the ZI? Does ZI_DA_ encode RPE as well? Do neuronal adaptations in the ZI drive drug intake, escalation, withdrawal symptoms, or compulsive seeking? Investigating these questions could clarify the ZI's role in addiction and its potential therapeutic relevance.

The ZI's potential role at the intersection between PD and drug addiction offers intriguing perspectives, not only for understanding its broader involvement in motor and psychiatric disorders, but also for its clinical relevance. ICDs, such as gambling, hypersexuality, or compulsive eating, are prevalent in PD (Houeto et al., 2016; Leclercq and Corvol, 2024), and resume most of the psychobiological mechanisms observed in drug addiction (Vassileva and Conrod, 2019). They are largely attributed to DA replacement therapy (Houeto et al., 2016), but their pathological underpinnings are still not fully characterized (Prange and Thobois, 2024), and might also involve ZI_DA_. Interestingly, L-DOPA enhances ZI neuronal activity in parkinsonian rats (Cole et al., 1993; Bastide et al., 2014), supporting this hypothesis.

ICDs are globally well alleviated by STN-DBS in patients with PD (Scherrer et al., 2020). Given the ZI's anatomical proximity to the STN and reports of compulsive behavior suppression following ZI-DBS (Mallet et al., 2002), this therapeutic benefit might partly involve modulation of ZI activity, particularly ZI_DA_. As DBS is increasingly applied to neurological disorders, including addiction (Vorspan et al., 2023; Pelloux and Baunez, 2013), and considering the clinical similarities between ICDs and drug addiction (Potenza, 2014), ZI-DBS may emerge as a potential therapeutic avenue for addictions. However, behavioral side effects associated with ZI-DBS (Ossowska, 2020) point out the need for major investigations to delineate ZI sectors and circuits potentially implicated in addiction and optimize therapeutic strategies.
